# The effects of different sowing methods on the yield and quality of cereal species in forage production

**DOI:** 10.7717/peerj.20640

**Published:** 2026-01-20

**Authors:** Hakan Kır

**Affiliations:** Department of Field Crops, Faculty of Agriculture, Kırşehir Ahi Evran University, Kırşehir, Turkey

**Keywords:** The perpendicular rows, Straight row, Dry matter yield, Crude protein

## Abstract

This research compares the yield and quality of barley (*Hordeum vulgare*), rye (*Secale cereale*), triticale (×*Triticosecale Wittmack*), and wheat (*Triticum aestivum*) grown in dry conditions in winter and widely used in producing quality forage using straight and perpendicular row sowing methods. The study was conducted during the 2019–2020 and 2020–2021 growing seasons using a randomized complete block design with split plots and three replications. Cereal species were placed in the main plots, while sowing methods were assigned to the subplots. Harvesting times were determined according to the flowering stages of the cereal species. According to the two-year study results, the highest dry matter yield was obtained from rye with the straight sowing method, while the lowest yield was obtained from barley using the same method. The best results for crude protein content, digestible dry matter content, and relative feed value were observed in barley; the lowest acid detergent fiber (ADF) and neutral detergent fiber (NDF) contents were recorded in rye. When evaluated regarding sowing methods, the perpendicular rows sowing method was superior to the straight row sowing method in yield. However, no significant effect of sowing methods on quality was observed. The perpendicular rows method of triticale is recommended for producing quality and high-yield forage in arid and semi-arid regions.

## Introduction

Cereals are the plant group with the largest cultivation area in the world, including Turkey. Since ancient times, this prevalence has been associated with the ease of transport, storage, and sale of cultivated cereals and their products. In addition to the vast diversity of species, varieties, and ecotypes among their cereals, their high adaptability further enhances their significance. The distinct differences among barley, wheat, rye, and triticale species and varieties enable these products to be evaluated based on their intended uses (Çaçan & Kökten, 2019; Helsel & Thomas, 1987). Because it has a shallow root system, barley is the cereal crop with the highest climatic and soil requirements among cool-season cereals (Çeri & Acar, 2019). Since it is resistant to salt and removes a significant amount from the soil, it is used in crop rotation in areas where irrigated agriculture is carried out. Barley is a preferred cereal crop for forage, and when harvested during the flowering stage, it is considered a highly digestible and high-quality feed (Cherney & Marten, 1982). Wheat (*Triticum aestivum* L.), generally grown for grain production, produces more feed than many spring cereal species. Livestock also prefers it due to its thin stems and high forage quality (Cash et al., 2007). Rye is a cereal crop with minimal soil and climate requirements. Rye’s strong root development enables it to utilize water and nutrients in the soil more efficiently, giving it a competitive advantage over other cool-season crops (Geçit, 2016). Suitable for sole and mixed cultivation with legumes, this plant provides quality feed when harvested during its flowering stage. The fact that there is no grain shedding during this period and that it does not consume too much water and nutrients from the soil increases its use in regions where water is limited (Geçit, 2016). Triticale, a hybrid of rye and wheat, is a suitable feed source for all types of livestock due to the low cost of its grain and vegetative parts. This feature makes triticale an alternative feed source. Triticale’s superior soil utilization efficiency compared with wheat, barley, and oats, along with its reduced susceptibility to environmental stresses, has increased demand for the crop in both sole and mixed cropping systems (Baron, Juskiw & Aljarrah, 2015). The reduced awn structure of triticale, compared to its ancestor rye, has made it more suitable for grazing and green fodder production as well as the development of varieties with even awn structure in recent years. Some researchers suggest that cool-climate cereals can be utilized as short-term pasture, silage, or hay playing a crucial role in sustainable livestock businesses by providing forage from winter to spring during the warm seasons when grass production is limited due to frost (Kim et al., 2017; Maloney, Oplinger & Albrecht, 1999). The variation in the intraspecific competition power of cereals, the higher tillering rate of barley compared to rye and oats, and the change in plant density according to the tillering rate, depending on the species, are essential for the effective use of available areas by plants. The row spacing of seeds determines the living space of the plants. Kaydan & Geçit (2005) stated that the seed amount and uniform distribution have a significant effect on yield. Baloch et al. (2002) stated that when plant density exceeds the optimum level, intra-species competition for light, water, and nutrients increases, leading to slower growth and decreased yield. In ecosystems with limited water availability, yield loss can be more pronounced. Moreover, the appropriate planting method can be used as an essential agricultural approach to increase yield, especially in cereals, by enhancing tillering capacity and making more efficient use of available resources (Hussain et al., 2012). The most used method in agricultural fields, straight-row sowing, is simpler and faster, making it preferred especially in large areas. On the other hand, perpendicular-row sowing is considered a more efficient and sustainable approach compared to straight-row sowing (Hussain et al., 2017). Given these considerations, sowing methods play a critical role in the economic and agronomic performance of cereal crops. When fuel and labor costs are high and crop market prices remain low, flat sowing is a more economically viable strategy. In contrast, under conditions of favorable crop prices, significant weed pressure, and potential yield gains, cross-sowing can serve as a more profitable alternative. The agronomic effectiveness of these methods, however, is influenced by site-specific factors such as local climatic conditions, soil properties, and the cultivated species. Consequently, a comprehensive evaluation of local conditions is essential for selecting the most appropriate sowing strategy Furthermore, studies on cereal species have demonstrated that cross-sowing suppresses weeds by providing a dense and homogeneous plant cover, thereby reducing herbicide use and energy costs, while simultaneously enhancing yield and sustainability (Gill & Walia, 2024; Hao et al., 2025; Hussain et al., 2012). Indeed, Dhatwalia et al. (2024) in their study on wheat where they tested broadcasting, line sowing, and criss-cross methods, found that the highest biological yield (9,357 kg/ha) was obtained with the criss-cross method. The researchers emphasized that the criss-cross method yields higher results, highlighting it as a more profitable approach. A study conducted by Suthar (2006) on wheat found that perpendicular rows sowing yielded significantly higher yields compared to line sowing, making it an effective sowing method. According to Kaydan & Geçit (2005), broadcasting provides the closest living space to the plant by ensuring a homogeneous distribution of seeds, while line sowing offers the longest rectangular area for the plant. In perpendicular sowing, the row spacing is 20 cm, similar to line sowing, and half of the seeds are sown in the line, while the other half is sown in the perpendicular rows (at 45° and 90°). In this case, the inter-row distances are doubled compared to line sowing, and the yields obtained from perpendicular sowing are higher than those from line sowing. In the perpendicular sowing method, the reduction in inter-row spaces significantly decreases weed biomass, which in turn reduces the nutrient consumption of weeds and increases yield (Frame, 2019; Kaydan et al., 2011). In a study by Boyd et al. (2009), which investigated the effects of different sowing methods and sowing rates on weed growth in rye, it was noted that the grid sowing arrangement did not provide a significant advantage. However, sowing rye at high rates and early in the season was found to increase dry matter production and reduce weed growth. It was concluded that while the grid arrangement is essential for weed suppression, the improvements it offers in rye dry matter production are relatively small. Thus, the grid sowing method is not advantageous compared to straight-row sowing. The suitability of both methods may vary depending on agricultural goals and the efficient use of available resources. In ecosystems with limited rainfall, experimenting with different cropping systems within the scope of sustainable farming practices is crucial. Such practices aim to increase the production of high-quality roughage and ensure the efficient use of natural resources. The province of Kırşehir faces a significant shortage of high-quality roughage, which limits the development of sustainable livestock production in the region. According to 2024 data from the Turkish Statistical Institute, the total small cattle population in the province is 419,000. This figure comprises 326,900 sheep, 23,000 goats, and 68,800 kids and lambs. Regarding large cattle, the total number stands at 278,000. This population includes 81,000 cows, 163,000 calves, and 33,000 bulls. In line with these data, the total animal population of Kırşehir Province has been determined to be 698,000 (Kır, 2024). The amount of quality roughage required by the current animal population is approximately 1,127 thousand tons. In Kırşehir, a total of roughly 106 thousand tons of quality roughage is produced from forage crop cultivation. In this case, only approximately 106 thousand tons of the province’s required roughage meet the demand of 1,127 thousand tons, and the remaining approximately 1,020 thousand tons cannot be met. According to this data, the rate of meeting Kırşehir’s quality roughage need is only 9.4%. Therefore, increasing the production of forage crops in field agriculture will significantly contribute to closing the roughage deficit in the region (Kır, 2024).

The provision of high-quality roughage is essential for sustainable livestock production, particularly in arid and semi-arid ecologies where water resources are scarce. This research was conducted to investigate the effects of different sowing methods on the yield and quality of specific cereal species, with the ultimate goal of offering viable solutions to the forage gap prevalent in comparable areas.

## Materials and Methods

The research was conducted in arid conditions during the 2019–2020 and 2020–2021 growing seasons at Kırşehir Ahi Evran University Bağbaşı Campus (1,090 m altitude, 39°08′N and 34°06′E).

The average temperature during the first (9.7 °C) and second (9.9 °C) growing seasons when the research was conducted was above the long-term average temperature (8.6 °C). In terms of relative humidity, the relative humidity rates obtained during both growing periods (62.4% and 59.2%) were below the long-term average relative humidity (68.3%) (Table 1). Similarly, the total precipitation recorded in the first growing season (317.4 mm) and the second growing season (255.2 mm) was below the long-term average of 356.1 mm (Table 1). Soil samples from the research area, prepared for sowing through soil tillage, were analyzed at the Kırşehir Ahi Evran University Central Research Laboratory Application and Research Center. According to the soil analysis results, the soil was classified as loamy (saturation 45.1), slightly alkaline (pH 8.1), non-saline (0.01), and highly calcareous (38.21), with a moderate level of available phosphorus (63 kg/ha) and rich in available potassium (1,485 kg/ha) the organic matter content of the soil was 1%.

**Figure 1:**
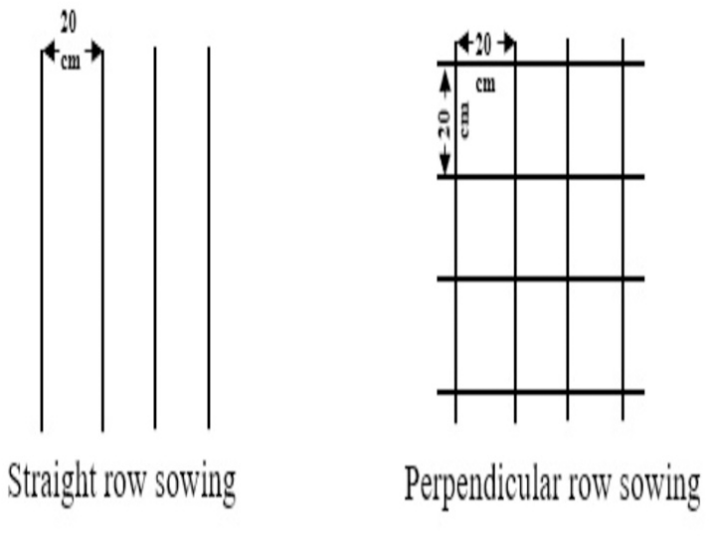
Sowing certain cereals in straight and perpendicular rows.

In this research, the plant materials chosen were Tarm-92 barley (*Hordeum vulgare*), Aslım-95 rye (*Secale cereale*), and Tatlıcak-97 triticale (×*Triticosecale Wittmack*), owing to their high suitability for the arid and semi-arid environments of Central Anatolia. Furthermore, the local Albostan wheat population (*Triticum aestivum*) was incorporated into the study given its specific adaptation to the climatic, edaphic, and agronomic conditions of the Kırşehir and Nevşehir provinces The experimental field was initially prepared by plowing to a depth of 15–20 cm to facilitate soil incorporation. Then, the trial area was cultivated perpendicular to the plowing direction using a cultivator and subsequently leveled. The soil tillage process was limited to avoid disrupting the soil structure and preserving the existing moisture. Since the cereal species were harvested during the flowering period and intended for fodder crop production, no control measures were required against weeds, diseases, or pests. The sowing methods involved two methods: sowing in straight rows and sowing in perpendicular rows, with the rows intersecting at a 90° angle. In the method of sowing in consecutive rows, sowing was carried out in 5-meter-long plots with rows spaced 20 cm apart, resulting in 10 rows per plot. In the method of sowing in perpendicular rows, both straight and perpendicular rows were created within the same 5-meter-long plot at 20 cm intervals (Fig. 1). Half of the seeds allocated for each plot were sown along the straight rows, and the other half along the perpendicular rows (Kaydan et al., 2011). In both sowing methods, the row spacing was maintained at 20 cm. The seeding density of cereals was kept at 500 seeds per square meter (Geçit, 2016). The experiment was established on 29 October 2019 in the first year and on 27 October 2020 in the second year.

**Table 1:** Climate data of Kirsehir province.

**Months** ^*^	**Precipitation (mm)**			**Relative Humidity (%)**			**Temperature (°C)**		
	**2019–20**	**2020–21**	**LTA**	**2019–20**	**2020–21**	**LTA**	**2019–20**	**2020–21**	**LTA**
October	1.1	9.1	30.4	52.8	39.8	62.7	16.0	17,1	13.1
November	30.4	20.3	41.6	60.6	64.1	72.4	8.5	6,5	6.3
December	61.9	17.5	47.1	80.8	73.0	79.0	3.9	4,6	2.0
January	42.0	40.8	44.3	71.2	71.0	79.0	1.2	3,5	−0.1
February	60.9	8.6	31.6	73.1	62.2	74.1	2.5	3,4	1.3
March	15.4	95.2	36.7	61.6	65.5	67.2	8.0	4,5	5.6
April	25.3	19.4	42.4	55.2	56.5	63.3	10.8	12,0	10.9
May	42.1	9.2	45.6	56.6	45.3	61.3	15.9	18,2	15.4
June	38.3	35.1	36.4	49.3	55.1	55.5	20.6	19,3	23.3
**Av./Total**	317.4	255.2	356.1	62.4	59.2	68.3	9.7	9.9	8.6

**Notes.**

* Turkish State Meteorological Service.

LTA Long-Term Average (1990–2020)

The research was established using a split-plot design in randomized blocks with three replications. The cereal species were placed in the main plots, while the sowing methods were placed in the subplots. Before sowing, 40 kg/ha of nitrogen (N) and 70 kg/ha of phosphorus (P_2_O_5_) were applied, while 30 kg/ha of nitrogen (N) was applied as a top fertilizer. The field trials for the research were established in the last week of October in both years. The harvest times were determined based on the flowering periods of the cereal species, starting with the earliest flowering species, and were conducted from 27 May to 7 June 2020 in the first year, and from 26 May to 7 July 2021 in the second year. During the harvesting process, one row from the edges of each plot and 50 cm from the ends of the plots were excluded as border effects, and the remaining area was harvested using a sickle (Göçmen & Parlak, 2017). The harvested plants were weighed to determine their green forage yield, and the necessary conversions were made to calculate the yields per hectare. 500 g of green forage samples were taken from the biomass harvested in each plot and dried at 60 °C until reaching a constant weight to determine the dry matter yield. The dry forage yields were then calculated using the obtained values (Sleugh et al., 2000). Then, the dried samples were ground through a one mm sieve for nutrient analysis. Acid Detergent Fiber (ADF) analysis is conducted by treating the sample with an acid detergent solution, which removes cell-soluble and hemicellulose. The residue that remains primarily consists of cellulose and lignin, components that are more resistant to microbial digestion in the rumen (Van Soest, Robertson & Lewis, 1991). In Neutral Detergent Fiber (NDF) analysis, the sample is treated with a neutral detergent solution that solubilizes cellular constituents, including proteins, sugars, lipids, and pectins. The remaining residue includes hemicellulose, cellulose, and lignin, which represent the structural components of the plant cell wall (Van Soest, Robertson & Lewis, 1991). ADF and NDF contents were determined using the ANKOM200 Fiber Analyzer, following the methodology developed by Van Soest, Robertson & Lewis (1991) and ANKOM Technology (2024). The nitrogen contents of the cereal species were determined using the Kjeldahl method, and these values were multiplied by a factor of 6.25 to obtain the crude protein contents (AOAC, 2005). According to the equation described by Sheaffer et al. (1995), digestible dry matter (DDM) was calculated as DDM = 88.9 −(0.779 ×% ADF (Table 1).

### Statistical analysis

The research data were subjected to analysis of variance (ANOVA) using the MSTAT-C statistical package. Duncan’s multiple range test was used for multiple comparisons, and the LSD test was applied for pairwise comparisons at *p* ≤ 0.05 (Petersen, 1994).

## Results

Dry matter yield differed significantly among cereal species in the first year of the research. Additionally, the differences among cereal species and sowing methods in the second year and the combined averages of both years were statistically significant (Table 2). According to the two-year average results, the dry matter yield was calculated as 5,757.0 kg/ha. The highest hay yield was obtained from rye (7,408.7 kg/ha), followed by lower yields from barley (4,849.8 kg/ha) and wheat (4,640.3 kg/ha). Regarding sowing methods, perpendicular sowing (6,082.6 kg/ha) provided a higher dry matter yield than straight-row sowing (5,431.3 kg/ha) (Table 2). In the results of the second year of the research and the two-year averages, all cereal species except rye differed statistically between straight row and perpendicular sowing methods. This interaction between the species and sowing method was statistically significant (Table 2).

**Table 2:** Dry matter yield (kg/ha) of some cereal species under different sowing methods.

**Species**	**Sowing method**	**2019–20**		**2020–21**		**Averages**	
**Barley**	Straight rows	4,155.3	±48.1	4,475.3	±315.7^c^^*^	4,315.3	±267.2^d^^**^
	Perpendicular rows	5,320.7	±358.5	5,448.0	±164.2^b^	5,384.3	±259.0^c^
	Average	4,738.0	±677.8^C+^	4,961.7	±578.5^C+^	4,849.8	±612.0^C+^
**Wheat**	Straight rows	4,531.3	±175.1	4,125.3	±275.1^c^	4,328.3	±303.4^d^
	Perpendicular rows	4,866.0	±123.5	5,038.3	±581.7^b^	4,952.2	±387.8^c^
	Average	4,698.7	±227.9^C^	4,581.8	±644.7^C^	4,640.3	±465.0^C^
**Rye**	Straight rows	7,715.7	±1,305.2	7,429.3	±61.4^a^	7,572.5	±841.1^a^
	Perpendicular rows	6,967.7	±716.2	7,522.0	±388.8^a^	7,244.8	±598.1^ab^
	Average	7,341.7	±1,026.9^A^	7,475.7	±254.0^A^	7,408.7	±716.6^A^
**Triticale**	Straight rows	5,627.0	±173.7	5,391.3	±326.8^b^	5,509.2	±267.3^c^
	Perpendicular rows	6,397.7	±546.1	7,100.7	±134.1^a^	6,749.2	±524.0^b^
	Average	6,012.3	±556.4^B^	6,246.0	±962.3^B^	6,129.2	±759.3^B^
**Average**	Straight rows	5,507.3	±1,553.4	5,355.3	±1,360.0^*B*^	5,431.3	±1,429.9^*B*^
	Perpendicular rows	5,888.0	±967.0	6,277.2	±1,144.9^*A*1^	6,082.6	±1,055.3^*A*1^
	Average	5,697.7	±1,280.3	5,816.3	±1,316.5	5,757.0	±1,286.0
**CV**		10.13	%	5.13	%	7.98	%

**Notes.**

** Differences between the averages followed by the same letter are not significant at *P* < 0.01 level.

* Differences between the averages followed by the same letter are not significant at *P* < 0.05 level.

+,1,2 Differences between the averages followed by the same letter are not significant at *P* < 0.01 level.

When evaluating both years of research and the combined averages, it was determined that cereal species and sowing methods caused statistically significant differences in crude protein contents. According to the data and averages of both years, the highest protein content was found in barley (140 g/kg, 118 g/kg, 129 g/kg), while the lowest protein content was obtained from rye (88 g/kg, 92 g/kg, 90 g/kg (Table 3). When the average crude protein contents of the two years were evaluated, values of 113 g/kg were obtained in straight sowing and 112 g/kg in perpendicular sowing. The crude protein content in the first year of the research (120 g/kg was higher than in the second year (104 g/kg rye was in the same statistical group for both straight and perpendicular sowing in the first year but in different groups in the second year. This indicates that the species × sowing method interaction was significant. While wheat and rye species were in different groups in the first year of the research, their presence in the same groups in the second year and the two-year averages resulted in a significant year × species interaction. In the first year, straight sowing (123 g/kg) provided a higher crude protein content than perpendicular sowing (117 g/kg) In contrast, in the second year, perpendicular sowing (106 g/kg) resulted in higher values than straight sowing (102 g/kg) (Table 3). This reveals that the year × sowing method interaction was significant. The variations in crude protein contents of rye across different years and sowing methods were caused by the year × sowing method × species interaction.

**Table 3:** Crude protein contents (g/kg) of some cereal species under different sowing methods.

**Species**	**Sowing method**	**2019–20**		**2020–21**		**Averages**	
**Barley**	Straight rows	144	±4.8^a^^*^	118	±7.7^a^^**^	131	±15.5
	Perpendicular rows	135	±4.3^b^	118	±5.3^a^	127	±10.6
	Average	140	±6.3^A+^	118	±5.9^A^^**^	129	±12.9^A+^
**Wheat**	Straight rows	121	±3.6^c^	105	±5.6^b^	113	±9.8
	Perpendicular rows	122	±3.8^c^	103	±2.7^bc^	112	±10.7
	Average	122	±3.3^C^	104	±4.1^B^	113	±9.8^B^
**Rye**	Straight rows	89	±4.0^d^	88	±1.9^e^	89	±2.9
	Perpendicular rows	86	±2.9^d^	95	±4.8^d^	91	±6.1
	Average	88	±3.4^D^	92	±5.3^C^	90	±4.7^C^
**Triticale**	Straight rows	138	±6.9^ab^	98	±5.0^cd^	118	±22.5
	Perpendicular rows	124	±1.1^c^	108	±1.7^b^	116	± 8.7
	Average	131	±8.8^B^	103	±6.5^B^	117	±16.3^B^
**Average**	Straight rows	123	±22.9^*A*1^	102	±12.3^*B*1^	113	±20.9
	Perpendicular rows	117	±19.3^*B*^	106	±9.1^*A*^	112	±15.8
	Average	120	±20.9^A2^	104	±10.8^B^	112	±18.3
**CV**		2.86	%	3.23	%	3.03	%

**Notes.**

** Differences between the averages followed by the same letter are not significant at *P* < 0.01 level.

* Differences between the averages followed by the same letter are not significant at *P* < 0.05 level.

+,1,2 Differences between the averages followed by the same letter are not significant at *P* < 0.01 level.

Regarding ADF and NDF, the differences among cereal species were significant in the first year, the second year, and the two-year averages of the research. In contrast, the differences among sowing methods were significant only in the second year of the study (Tables 4 and 5). According to the two-year average results, ADF was 316 g/kg, while NDF was 506 g/kg. The lowest ADF ratio (265 g/kg) was obtained from barley, barley (459 g/kg) and triticale (472 g/kg) formed the statistically lowest group for NDF. Rye had the highest ADF and NDF ratios, with values of 377 g/kg and 571 g/kg, respectively. In the second year of the research, the ADF ratio obtained from perpendicular sowing (308 g/kg) was statistically higher than that obtained from straight sowing (301 g/kg). In the two-year averages, the ADF ratio was determined to be 313 g/kg,and the NDF ratio was 504 g/kg in straight sowing, while the ADF ratio was 320 g/kg and the NDF ratio was 509 g/kg in perpendicular sowing. In the second year of the research, differences in ADF ratios for wheat and NDF ratios for barley were observed depending on sowing methods, leading to significant interaction between species and sowing methods. The higher ADF and NDF ratios obtained in the first year of the research compared to the second year indicated that the year factor was significant. Regarding ADF ratios, wheat and rye, which were in the same group in the second year of the research, were placed in different groups in the two-year averages (Table 4). Regarding NDF ratios, barley and wheat were in the same group in the first year and placed in different groups in the two-year averages (Table 5).

**Table 4:** Acid detergent fiber (g/kg) of some cereal species under different sowing methods.

**Species**	**Sowing method**	2019–20		2020–21		**Averages**	
**Barley**	Straight rows	278	±21.5	237	±2.7^d^^**^	258	±26.0^f^^**^
	Perpendicular rows	297	±20.9	247	±9.2d	272	±31.2^ef^
	Average	288	±21.7^C+^	242	±7.8^C+^	265	±28.3^D+^
**Wheat**	Straight rows	328	±18.9	359	±3.9^a^	344	±21.0^bc^
	Perpendicular rows	330	±3.3	331	±1.6^b^	331	±2.5^c^
	Average	329	±12.1^B^	345	±15.5^A^	337	±15.8^B^
**Rye**	Straight rows	394	±17.5	327	±9.1^b^	360	±39.0^b^
	Perpendicular rows	418	±8.8	368	±5.0^a^	393	±28.1^a^
	Average	406	±18.1^A^	348	±23.7^A^	377	±36.7^A^
**Triticale**	Straight rows	302	±2.4	280	±6.7^c^	291	±13.0^d^
	Perpendicular rows	281	±9.5	285	±4.0^c^	283	±6.8^de^
	Average	292	±13.2^C^	282	±5.5^B^	287	±10.8^C^
**Average**	Straight rows	326	±47.6	301	±48.5^*B*^	313	±48.6
	Perpendicular rows	331	±56.4	308	±48.4^*A*^^1^	320	±52.8
	Average	329	±51.1^A2^	304	±47.5^B^	316	±50.3
**CV**		5.70	%	1.81	%	4.36	%

**Notes.**

** Differences between the averages followed by the same letter are not significant at *P* < 0.01 level.

+,1,2 Differences between the averages followed by the same letter are not significant at *P* < 0.01 level.

**Table 5:** Neutral detergent fiber (g/kg) of some cereal species under different sowing methods.

**Species**	**Sowing method**	**2019–20**		**2020–21**		**Averages**	
**Barley**	Straight rows	496	±53.6	432	±3.4^d^^*^	464	±48.4
	Perpendicular rows	499	±14.6	408	±1.8^e^	454	±50.8
	Average	498	±35.2^B+^	420	±13.3^D+^	459	±47.6^C+^
**Wheat**	Straight rows	503	±21.0	534	±6.3^a^	519	±21.8
	Perpendicular rows	522	±15.5	532	±2.3^a^	527	±11.5
	Average	513	±19.4^B^	533	±4.3^A^	523	±17.2^B^
**Rye**	Straight rows	624	±17.2	503	±8.3^b^	564	±67.3
	Perpendicular rows	647	±10.7	511	±11.2^b^	579	±74.9
	Average	636	±18.0^A^	507	±9.9^B^	571	±68.4^A^
**Triticale**	Straight rows	481	±3.4	459	±5.8^c^	470	±12.9
	Perpendicular rows	486	±13.7	462	±4.3^c^	474	±15.7
	Average	484	±9.2^B^	461	±4.8^C^	472	±13.9^C^
**Average**	Straight rows	526	±65.0	482	±41.3	504	±57.8
	Perpendicular rows	539	±67.9	479	±50.2	509	±65.9
	Average	532	±65.3^A1^	480	±45.0^B^	506	±61.4
**CV**		4.97	%	1.50	%	3.83	%

**Notes.**

* Differences between the averages followed by the same letter are not significant at *P* < 0.05 level.

+,1 Differences between the averages followed by the same letter are not significant at *P* < 0.01 level.

This demonstrates that the year × species interaction was significant. In the research, differences among cereal species regarding digestible dry matter (DDM) yield were statistically significant for the first year, the second year, and the combined averages of the two years (Table 6). The differences between sowing methods were significant only in the second year. The two-year average results show that the mean DDM ratio was 643 g/kg. Within the cereal species, the highest DDM ratio was obtained from barley at 683 g/kg, while the lowest was obtained from rye at 596 g/kg. In the second year of the research, the DDM ratio obtained through straight sowing (655 g/kg was statistically higher than that obtained through perpendicular sowing (649 g/kg). When the two-year averages were examined, the DDM ratio was determined to be 645 g/kg in straight sowing and 640 g/kg in perpendicular sowing. Generally, considering the cereal species and sowing methods, the average DDM ratio was 643 g/kg. In the second year and the two-year averages, the species × sowing method interaction was found to be statistically significant as different results for rye between straight and perpendicular sowing methods. In the first year of the research, the DDM ratio was 633 g/kg, while in the second year, it increased to 652 g/kg. This increase was found to be statistically significant. Rye and wheat were in the same group in the second but were placed in different statistical groups in the first year and the two-year averages. This indicates that the year × species interaction is significant (Table 6).

**Table 6:** Digestible dry matter ratio (g/kg) of some cereal species under different sowing methods.

**Species**	**Sowing method**	**2019–20**		**2020–21**		**Averages**	
**Barley**	Straight rows	673	±16.8	704	±2.1^a^^**^	688	±20.2^a^^**^
	Perpendicular rows	658	±16.3	697	±7.2^a^	677	±24.3^ab^
	Average	665	±16.9^A+^	701	±6.1^A+^	683	±22.1^A+^
**Wheat**	Straight rows	634	±14.7	609	±3.0^d^	621	±16.3^de^
	Perpendicular rows	632	±2.6	631	±1.2^c^	632	±2.0^d^
	Average	633	±9.5^B^	620	±12.0^C^	627	±12.3^C^
**Rye**	Straight rows	582	±13.6	634	±7.1^c^	608	±30.4^e^
	Perpendicular rows	563	±6.9	602	±3.9^d^	583	±21.9^f^
	Average	573	±14.1^C^	618	±18.4^C^	596	±28.6^D^
**Triticale**	Straight rows	654	±1.9	671	±5.2^b^	662	±10.1^c^
	Perpendicular rows	670	±7.4	667	±3.1^b^	669	±5.3^bc^
	Average	661	±10.3^A^	669	±4.3^B^	666	±8.4^B^
**Average**	Straight rows	636	±37.1	655	±37.8^*A*^^1^	645	±37.9
	Perpendicular rows	631	±43.9	649	±37.7^*B*^	640	±41.1
	Average	633	±39.8^B2^	652	±37.0^A^	643	±39.2
**CV**		2.43	%	0.64	%	1.70	%

**Notes.**

** Differences between the averages followed by the same letter are not significant at *P* < 0.01 level.

+,1,2 Differences between the averages followed by the same letter are not significant at *P* < 0.01 level.

## Discussion

Determining the appropriate sowing method is crucial for identifying suitable species and achieving the highest yield from these species. Across the different cereal species with various sowing methods, rye (7,408.7 kg/ha) was the cereal with the highest dry matter yield, while lower yields were obtained from barley (4,849.8 kg/ha) and wheat (464.30 kg/ha). Rye utilizes soil nutrients more effectively due to its vigorous root development. Rye’s earlier growth and development in spring, and its more efficient use of spring rainfall, positively influence vegetative growth (Geçit, 2016). In the research, the rapid tillering of the plant due to intraspecific competition along with its ability to produce tall plants, contributed to an increase in the dry matter yield of rye (Çeri & Acar, 2019). Kaspar & Bakker (2015) and Liebert et al. (2023) stated that rye yields more than triticale and barley. The root system of barley is more superficial than that of other cereal species, which limits its ability to benefit sufficiently from water and nutrients deep in the soil. Regarding nutrient requirements, barley is followed by wheat. The average dry matter yields obtained from different wheat and barley sowing methods were placed in the statistically lower group compared with triticale and oat. Rye has provided a high dry matter yield through straight sowing (7,572.5 kg/ha) and perpendicular rows (7,244.8 kg/ha). It can be said that intra-row competition in rye plants is more pronounced than inter-row competition. Unlike other cereals, the straight sowing method can explain this situation by achieving higher yields. Due to intraspecific competition, the plants’ need for sunlight to perform more photosynthesis has increased plant height, contributing to higher yields. Additionally, largely unaffected by changes in the area per plant, rye plants have achieved the highest dry matter yield by efficiently utilizing available resources despite these limitations. Due to the low intraspecific competition in wheat and barley, lower dry matter yields (4,328.3 kg/ha and 4,315.3 kg/ha, respectively) were obtained in straight sowing. The average dry matter yield across different sowing methods for cereal species was 5,757.0 kg/ha, with perpendicular rows providing 6,082.6 kg/ha and straight sowing yielding 5,431.3 kg/ha. In the perpendicular rows of cereal species with a row spacing of 20 cm, sowing half of the seed amount in the first row and the other half in the cross row (90°) for a better evaluation of the environmental conditions, provided higher yields. Kaydan et al. (2011), stated that the perpendicular rows method reduces weed biomass and increases yield. It is known that forage quality in cereals is affected by various factors, including the cereal species and varieties used, the harvest period, and the agricultural methods employed. A high crude protein content is a desirable characteristic for good forage quality. In the study, barley grown using different sowing methods had the highest crude protein content (129 g/kg, according to Cherney & Marten (1982). barley harvested at the flowering stage has high forage quality due to its digestibility. The study found that the lowest crude protein content was obtained from rye, 90 g/kg of dry matter. According to Lacefield (1988) states that the quality standards of all cereal species range between 110–130 g/kg and are classified as medium level. Francia et al. (2006) determined the protein content of barley and oat forage during the vegetative stage as 234 g/kg and 245 g/kg, respectively, However, they observed that these contents decreased to 6% and 7% during the dough stage. De Ruiter et al. (2002) reported that the protein content in cereal forage varied between 10% and 30% depending on the harvesting stage. They determined that the protein content in triticale harvested during the vegetative stage ranged between 17.9 g/kg and 26.1 g/kg. In the first year of the study, the average crude protein content of species grown using straight sowing methods (123 g/kg) was higher than that of species grown using perpendicular row methods (117 g/kg). In the second year, however, the average crude protein content in straight sowing (102 g/kg was lower than that in perpendicular rows (106 g/kg). The higher rainfall in the first year compared to the second year contributed to increased crude protein content in straight sowing. In the second year, with increased intraspecific competition, the perpendicular rows method enabled a more efficient use of environmental resources through root competition, resulting in a higher crude protein content in perpendicular rows (Table 1). Andrew, Storkey & Sparkes (2015) stated root competition would be more dominant than light competition. In the first year of the study, higher rainfall likely contributed to an increase in average crude protein content. This effect may be attributed to enhanced leaf growth and higher leaf-to-stem ratios associated with greater water availability, which in turn can promote higher crude protein levels in the plant. These results suggest that variations in water supply can indirectly influence forage quality through their impact on plant growth dynamics. Indeed, Moore & Undersander (2002) also stated that a higher leaf ratio correlates with higher protein content, emphasizing that high-quality forages have a high leaf ratio and protein content (Moore, Kunkle & Brown, 1991). Although yield is the most important criterion for livestock enterprises, in recent years, great importance has been placed on feed quality and the nutritional value of feed. Cereals have higher levels of cell wall components compared to legumes. The cell wall constituent’s acid detergent fiber (ADF) and neutral detergent fiber (NDF) are key indicators of forage intake and digestibility. Consequently, determining the ADF and NDF contents in cereals is of significant importance. In the study, rye had the highest values in ADF and NDF rates among the species grown using different sowing methods, while barley and triticale had lower values and formed the lower group. This may be because rye contains more cell wall components than other species. Higher ADF contents were obtained in the perpendicular rows of barley and rye compared to straight sowing. In contrast, higher ADF contents were observed in the straight sowing of wheat and triticale compared to perpendicular rows. The intraspecific competition, tillering characteristics, and ways of utilizing the environmental conditions of the cereal species used in the study may have contributed to the differences observed between the sowing methods. Additionally, in regions like Central Anatolia, where temperature and water stress are prevalent, plants with access to water may tend to utilize more sunlight, which could contribute to an increase in their height and the proportion of cell wall components. Among the species examined, barley had the highest digestible dry matter content, while rye had the lowest. Cherney & Marten (1982) stated that barley harvested during the flowering stage has high digestibility.

Niemann et al. (1992) and Poorter & Bergkotte (1992) stated that the accumulation of nitrogenous compounds, which are associated with cytoplasmic components, is higher in rapidly growing species. In contrast, slowly growing species exhibit a greater accumulation of cell wall components, such as lignin, cellulose, hemicellulose, and other water-insoluble structural carbohydrates. However, in the study, rye’s better utilization of environmental conditions in early spring and its higher cell wall content than other cereal species influenced the ADF levels. The increased ADF levels negatively affected the digestible dry matter ratios. As a result, different findings have been obtained from the results of Niemann et al. (1992) and Poorter & Bergkotte (1992). Because of the inverse relationship between digestible dry matter ratios and ADF levels, the DDM ratio in the second year was higher compared to the first year, as ADF levels were lower in the second year. Additionally, an inverse pattern was observed in DDM ratios relative to ADF levels with respect to sowing methods. In barley and rye, the straight sowing method provided higher DDM ratios than the perpendicular rows method. In contrast, in wheat and triticale, the perpendicular rows method resulted in higher DDM ratios than the straight sowing method. Indeed, according to Linn & Martin (1989), it is closely related to digestibility, and variations in ADF levels influence the digestibility of forage. Determining a forage crop’s chemical, physical, and biological properties is important for assessing its quality.

## Conclusion

The research results will serve as a guide in selecting cereal species and sowing methods to meet the forage needs of livestock enterprises and enhance the production of high-quality feed. In this context, sowing methods and species selection should be strategically evaluated to meet the forage needs of sustainable livestock enterprises. The results obtained from the research provide valuable contributions to sustainable agricultural practices that aim to protect and utilize natural resources efficiently. The effects of different sowing methods on the yield and quality of barley, wheat, rye, and triticale cereal species cultivated since ancient times and continuously evolving with mechanization were investigated. In the research, rye attracted attention with its high yield, while barley was notable for its superior forage quality. When yield and quality are evaluated, the perpendicular rows method provides advantages in both respects, especially for triticale. Perpendicular rows of triticale are recommended to support the production of high-quality forage in arid and semi-arid regions. In such regions, choosing species like triticale for forage production and properly planning sowing methods can significantly enhance productivity and forage quality. At the same time, these approaches can make a substantial contribution to sustainable agriculture by efficiently utilizing natural resources.

##  Supplemental Information

10.7717/peerj.20640/supp-1 Supplemental Information 1 Raw data Data are the averages taken from the randomized block design with three replications. This includes the data and averages for the years 2019-2020, 2020-2021 and average of years.

10.7717/peerj.20640/supp-2 Supplemental Information 2 Data after statistical analysis

## Additional Information and Declarations

### Data Availability

The following information was supplied regarding data availability: The raw data are available in the Supplemental Files.

## References

[ref-1] Andrew I, Storkey J, Sparkes D (2015). A review of the potential for competitive cereal cultivars as a tool in integrated weed management. Weed Research.

[ref-2] ANKOM Technology (2024). Analytical methods fiber analyzer A200. https://www.ankom.com/analytical-methods-support/fiber-analyzer-a200.

[ref-3] AOAC (2005). Official methods of analysis. Association of Official Analytical Chemists.

[ref-4] Baloch AW, Soomro AM, Javed MA, Ahmad M, Bughio HR, Bughio MS (2002). Optimum plant density for high yield in rice (*Oryza sativa* L.). Asian Journal of Plant Sciences.

[ref-5] Baron VS, Juskiw PE, Aljarrah M, Eudes F (2015). Triticale as a Forage. Triticale.

[ref-6] Boyd NS, Brennan EB, Smith RF, Yokota R (2009). Effect of seeding rate and planting arrangement on Rye cover crop and weed growth. Agronomy Journal.

[ref-7] Çaçan E, Kökten K (2019). A research on the evaluation of the cereal species as Roughage. Journal of Agriculture Faculty of Ege University.

[ref-8] Cash D, Carlstrom R, Surber L, Hafl A (2007). Forage yield and quality of ‘Willow Creek’ forage winter wheat.

[ref-9] Çeri S, Acar R (2019). Use of cool climate cereals as green and dry forage in animal feeding. Journal of Bahri Dagdas Crop Research.

[ref-10] Cherney J, Marten G (1982). Small grain crop forage potential: I. Biological and chemical determinants of quality, and yield^1^. Crop Science.

[ref-11] De Ruiter J, Hanson R, Hay A, Armstrong K, Harrison-Kirk R (2002). Whole-crop cereals for grazing and silage: balancing quality and quantity. Proceedings of the New Zealand Grassland Association.

[ref-12] Dhatwalia RK, Choudhary K, Bochalya RS, Devi R, Kumar R, Sharma A, Meenakshi, Sharma A (2024). Effect of different fertility levels and sowing methods on growth, yield and economics of wheat (*Triticum aestivum* l.). International Journal of Research in Agronomy.

[ref-13] Frame J (2019). Forage legumes for temperate grasslands. Food and Agriculture Organization of the United Nations.

[ref-14] Francia E, Pecchioni N, Nicosia OLD, Paoletta G, Taibi L, Franco V, Odoardi M, Stanca AM, Delogu G (2006). Dual-purpose barley and oat in a Mediterranean environment. Field Crops Research.

[ref-15] Geçit HH (2016). Cool climate cereals (wheat, barley, oats, triticale).

[ref-16] Gill HK, Walia US (2024). Effect of planting patterns and weed competition on growth, yield and quality of wheat (*Triticum aestivum* L.). Agricultural Science Digest.

[ref-17] Göçmen N, Parlak AÖ (2017). Determination of seeding raties of pea intercrops with oat, barley and triticale. COMU Journal of Agriculture Faculty.

[ref-18] Hao F, Yu T, Gao K, Xiong M, An H (2025). Production performance and stability of mixed forage grasslands improved by planting proportion and mode in Horqin sandy land, China. Scientific Reports.

[ref-19] Helsel ZR, Thomas JW (1987). Small grains for forage. Journal of Dairy Science.

[ref-20] Hussain I, Khan EA, Hassan G, Gul J, Ozturk M, Alharby H, Hakeem KR, Alamri S (2017). Integration of high seeding densities and criss cross row planting pattern suppresses weeds and increases grain yield of spring wheat. Journal of Environmental Biology.

[ref-21] Hussain M, Mehmood Z, Khan MB, Farooq S, Lee D-J, Farooq M (2012). Narrow row spacing ensures higher productivity of low tillering wheat cultivars. International Journal of Agriculture and Biology.

[ref-22] Kaspar TC, Bakker MG (2015). Biomass production of 12 winter cereal cover crop cultivars and their effect on subsequent no-till corn yield. Journal of Soil and Water Conservation.

[ref-23] Kaydan D, Geçit HH (2005). The effect of sowing methods and sowing densities on yield and yield components of Barley. Yuzuncu Yil University Journal of Agricultural Sciences.

[ref-24] Kaydan D, Tepe I, Yagmur M, Yergin R (2011). Effects of sowing methods and rates on weeds, grain yield and some yield components of wheat. Journal of Agricultural Sciences-Tarim Bilimleri Dergisi.

[ref-25] Kim KS, Anderson JD, Webb SL, Newell MA, Butler TJ (2017). Variation in winter forage production of four small grain species—oat, rye, triticale, and wheat. Pakistan Journal of Botany.

[ref-26] Kır H (2024). Roughage production and needs analysis in Türkiye: the example of the Tr 71 region. Scientific research in science, agriculture and engineering.

[ref-27] Lacefield GD (1988). Alfalfa hay quality makes the difference.

[ref-28] Liebert J, Cherney JH, Ketterings QM, Mirsky SB, Pelzer CJ, Ryan MR (2023). Winter cereal species, cultivar, and harvest timing affect trade-offs between forage quality and yield. Frontiers in Sustainable Food Systems.

[ref-29] Linn JG, Martin NP (1989). Forage quality tests and interpretation.

[ref-30] Maloney TS, Oplinger ES, Albrecht KA (1999). Small grains for fall and spring forage. Journal of Production Agriculture.

[ref-31] Moore JE, Kunkle WE, Brown WF (1991). Forage quality and the need for protein and energy supplements.

[ref-32] Moore JE, Undersander DJ (2002). Relative forage quality: an alternative to relative feed value and quality index.

[ref-33] Niemann GJ, Pureveen JB, Eijkel GB, Poorter H, Boon JJ (1992). Differences in relative growth rate in 11 grasses correlate with differences in chemical composition as determined by pyrolysis mass spectrometry. Oecologia.

[ref-34] Petersen RG (1994). Agricultural field experiments.

[ref-35] Poorter H, Bergkotte M (1992). Chemical-composition of 24 wild-species differing in relative growth-rate. Plant Cell and Environment.

[ref-36] Sheaffer C, Peterson M, McCaslin M, Volenec J, Cherney J, Johnson K, Woodward W, Viands D (1995). Acid detergent fiber, neutral detergent fiber concentration and relative feed value. StandArt tests to characterize alfalfa cultivars.

[ref-37] Sleugh B, Moore KJ, George JR, Brummer EC (2000). Binary legume-grass mixtures improve forage yield, quality, and seasonal distribution. Agronomy Journal.

[ref-38] Suthar SL (2006). Effect of sowing methods, nitrogen and chemical weed control on wheat (*Triticum aestivum* l.).

[ref-39] Van Soest PJ, Robertson JB, Lewis BA (1991). Methods for dietary fiber, neutral detergent fiber, and nonstarch polysaccharides in relation to animal nutrition. Journal of Dairy Science.

