# Supplementary material for: The effects of different sowing methods on the yield and quality of cereal species in forage production
Source: PeerJ. 2026 Jan 20;14:e20640. doi: 10.7717/peerj.20640 (PMC12829460; doi:10.7717/peerj.20640)
Supplement: Supplemental Information 2 [file peerj-14-20640-s002.docx]

Yield and Quality Characteristics of Some Cereal Species Under Different Sowing Methods

| **Species** | Sowing Method | **Dry Matter Yield (kg/da)** | | | | | | | | | | | | | | | | | **Crude Protein Ratio (%)** | | | | | | | | | | | | | | | |
| --- | --- | --- | --- | --- | --- | --- | --- | --- | --- | --- | --- | --- | --- | --- | --- | --- | --- | --- | --- | --- | --- | --- | --- | --- | --- | --- | --- | --- | --- | --- | --- | --- | --- | --- |
|  |  | 2019-20 | | | | | | 2020-21 | | | | | **Averages** | | | | | | 2019-20 | | | | | | | 2020-21 | | | | | | **Averages** | | |
| **Barley** | Straight Rows | 415.5 | |  | | | | 447.5 | | | c^*^ | | 431.5 | | | d^**^ | | | 14.4 | | | a^*^ | | | | 11.8 | | | a^**^ | | | 13.1 |  | |
|  | Perpendicular Rows | 532.1 | |  | | | | 544.8 | | | b | | 538.4 | | | c | | | 13.5 | | | b | | | | 11.8 | | | a | | | 12.7 |  | |
|  | Average | 473.8 | | C^+^ | | | | 496.2 | | | C^+^ | | 485.0 | | | C^+^ | | | 14.0 | | | A^+^ | | | | 11.8 | | | A^**^ | | | 12.9 | A^+^ | |
| **Wheat** | Straight Rows | 453.1 | |  | | | | 412.5 | | | c | | 432.8 | | | d | | | 12.1 | | | c | | | | 10.5 | | | b | | | 11.3 |  | |
|  | Perpendicular Rows | 486.6 | |  | | | | 503.8 | | | b | | 495.2 | | | c | | | 12.2 | | | c | | | | 10.3 | | | bc | | | 11.2 |  | |
|  | Average | 469.9 | | C | | | | 458.2 | | | C | | 464.0 | | | C | | | 12.2 | | | C | | | | 10.4 | | | B | | | 11.3 | B | |
| **Rye** | Straight Rows | 771.6 | |  | | | | 742.9 | | | a | | 757.3 | | | a | | | 8.9 | | | d | | | | 8.8 | | | e | | | 8.9 |  | |
|  | Perpendicular Rows | 696.8 | |  | | | | 752.2 | | | a | | 724.5 | | | ab | | | 8.6 | | | d | | | | 9.5 | | | d | | | 9.1 |  | |
|  | Average | 734.2 | | A | | | | 747.6 | | | A | | 740.9 | | | A | | | 8.8 | | | D | | | | 9.2 | | | C | | | 9.0 | C | |
| **Triticale** | Straight Rows | 562.7 | |  | | | | 539.1 | | | b | | 550.9 | | | c | | | 13.8 | | | ab | | | | 9.8 | | | cd | | | 11.8 |  | |
|  | Perpendicular Rows | 639.8 | |  | | | | 710.1 | | | a | | 674.9 | | | b | | | 12.4 | | | c | | | | 10.8 | | | b | | | 11.6 |  | |
|  | Average | 601.2 | | B | | | | 624.6 | | | B | | 612.9 | | | B | | | 13.1 | | | B | | | | 10.3 | | | B | | | 11.7 | B | |
| **Average** | Straight Rows | 550.7 | |  | | | | 535.5 | | | *B* | | 543.1 | | | *B* | | | 12.3 | | | *A^1^* | | | | 10.2 | | | *B^1^* | | | 11.3 |  | |
|  | Perpendicular Rows | 588.8 | |  | | | | 627.7 | | | *A^1^* | | 608.3 | | | A*^1^* | | | 11.7 | | | *B* | | | | 10.6 | | | *A* | | | 11.2 |  | |
|  | Average | 569.8 | |  | | | | 581.6 | | |  | | 575.7 | | |  | | | **12.0** | | | **A^2^** | | | | **10.4** | | | **B** | | | 11.2 |  | |
| **CV** | | 10.13 | | % | | | | 5.13 | | | % | | 7.98 | | | % | | | 2.86 | | | % | | | | 3.23 | | | % | | | 3.03 | % | |
| Species | Sowing Method | **Acid Detergent Fiber (%)** | | | | | | | | | | | | | | | | | | **Neutral Detergent Fiber (%)** | | | | | | | | | | | | | | |
|  |  | 2019-20 | | | | | 2020-21 | | | | | | | **Averages** | | | | | | 2019-20 | | | | | 2020-21 | | | | | | **Averages** | | | |
| **Barley** | Straight Rows | 27.8 | | |  | | 23.7 | | | d^**^ | | | | 25.8 | | | f ^**^ | | | 49.6 | | |  | | 43.2 | | | d^*^ | | | 46.4 | | |  |
|  | Perpendicular Rows | 29.7 | | |  | | 24.7 | | | d | | | | 27.2 | | | ef | | | 49.9 | | |  | | 40.8 | | | e | | | 45.4 | | |  |
|  | Average | 28.8 | | | C^+^ | | 24.2 | | | C^+^ | | | | 26.5 | | | D^+^ | | | 49.8 | | | B^+^ | | 42.0 | | | D^+^ | | | 45.9 | | | C^+^ |
| **Wheat** | Straight Rows | 32.8 | | |  | | 35.9 | | | a | | | | 34.4 | | | bc | | | 50.3 | | |  | | 53.4 | | | a | | | 51.9 | | |  |
|  | Perpendicular Rows | 33.0 | | |  | | 33.1 | | | b | | | | 33.1 | | | c | | | 52.2 | | |  | | 53.2 | | | a | | | 52.7 | | |  |
|  | Average | 32.9 | | | B | | 34.5 | | | A | | | | 33.7 | | | B | | | 51.3 | | | B | | 53.3 | | | A | | | 52.3 | | | B |
| **Rye** | Straight Rows | 39.4 | | |  | | 32.7 | | | b | | | | 36.0 | | | b | | | 62.4 | | |  | | 50.3 | | | b | | | 56.4 | | |  |
|  | Perpendicular Rows | 41.8 | | |  | | 36.8 | | | a | | | | 39.3 | | | a | | | 64.7 | | |  | | 51.1 | | | b | | | 57.9 | | |  |
|  | Average | 40.6 | | | A | | 34.8 | | | A | | | | 37.7 | | | A | | | 63.6 | | | A | | 50.7 | | | B | | | 57.1 | | | A |
| **Triticale** | Straight Rows | 30.2 | | |  | | 28.0 | | | c | | | | 29.1 | | | d | | | 48.1 | | |  | | 45.9 | | | c | | | 47.0 | | |  |
|  | Perpendicular Rows | 28.1 | | |  | | 28.5 | | | c | | | | 28.3 | | | de | | | 48.6 | | |  | | 46.2 | | | c | | | 47.4 | | |  |
|  | Average | 29.2 | | | C | | 28.2 | | | B | | | | 28.7 | | | C | | | 48.4 | | | B | | 46.1 | | | C | | | 47.2 | | | C |
| **Average** | Straight Rows | 32.6 | | |  | | 30.1 | | | *B* | | | | 31.3 | | |  | | | 52.6 | | |  | | 48.2 | | |  | | | 50.4 | | |  |
|  | Perpendicular Rows | 33.1 | | |  | | 30.8 | | | *A*^1^ | | | | 32.0 | | |  | | | 53.9 | | |  | | 47.9 | | |  | | | 50.9 | | |  |
|  | Average | 32.9 | | | A^2^ | | 30.4 | | | B | | | | 31.6 | | |  | | | 53.2 | | | A^2^ | | 48.0 | | | B | | | 50.6 | | |  |
| **CV** | | 5.70 | | | % | | 1.81 | | | % | | | | 4.36 | | | % | | | 4.97 | | | % | | 1.50 | | | % | | | 3.83 | | | % |
| **Species** | Sowing Method | **Digestible Dry Matter Ratio (%)** | | | | | | | | | | | | | | | | **Relative Feed Value** | | | | | | | | | | | | | | | | |
|  |  | 2019-20 | | | | 2020-21 | | | | | | **Averages** | | | | | | 2019-20 | | | | | | 2020-21 | | | | | | **Averages** | | | | |
| **Barley** | Straight Rows | 67.3 |  | | | 70.4 | | | a^**^ | | | 68.8 | | | a^**^ | | | 127.5 | | |  | | | 151.5 | | | b^*^ * | | | 139.5 | | | |  |
|  | Perpendicular Rows | 65.8 |  | | | 69.7 | | | a | | | 67.7 | | | ab | | | 122.6 | | |  | | | 158.8 | | | a | | | 140.7 | | | |  |
|  | Average | 66.5 | A^+^ | | | 70.1 | | | A^+^ | | | 68.3 | | | A^+^ | | | 125.1 | | | A^**^ | | | 155.1 | | | A^+^ | | | 140.1 | | | | A^+^ |
| **Wheat** | Straight Rows | 63.4 |  | | | 60.9 | | | d | | | 62.1 | | | de | | | 117.2 | | |  | | | 106.1 | | | f | | | 111.7 | | | |  |
|  | Perpendicular Rows | 63.2 |  | | | 63.1 | | | c | | | 63.2 | | | d | | | 112.8 | | |  | | | 110.2 | | | e | | | 111.5 | | | |  |
|  | Average | 63.3 | B | | | 62.0 | | | C | | | 62.7 | | | C | | | 115.0 | | | B | | | 108.2 | | | D | | | 111.6 | | | | C |
| **Rye** | Straight Rows | 58.2 |  | | | 63.43 | | | c | | | 60.8 | | | e | | | 86.8 | | |  | | | 117.3 | | | d | | | 102.1 | | | |  |
|  | Perpendicular Rows | 56.3 |  | | | 60.23 | | | d | | | 58.3 | | | f | | | 81.0 | | |  | | | 109.5 | | | ef | | | 95.3 | | | |  |
|  | Average | 57.3 | C | | | 61.8 | | | C | | | 59.6 | | | D | | | 83.9 | | | C | | | 113.4 | | | C | | | 98.7 | | | | D |
| **Triticale** | Straight Rows | 65.4 |  | | | 67.1 | | | b | | | 66.2 | | | c | | | 126.2 | | |  | | | 135.9 | | | c | | | 131.1 | | | |  |
|  | Perpendicular Rows | 67.0 |  | | | 66.7 | | | b | | | 66.9 | | | bc | | | 128.4 | | |  | | | 134.3 | | | c | | | 131.4 | | | |  |
|  | Average | 66.1 | A | | | 66.9 | | | B | | | 66.6 | | | B | | | 127.3 | | | A | | | 135.1 | | | B | | | 131.2 | | | | B |
| **Average** | Straight Rows | 63.6 |  | | | 65.5 | | | *A*^1^ | | | 64.5 | | |  | | | 114.4 | | |  | | | 127.7 | | |  | | | 121.1 | | | |  |
|  | Perpendicular Rows | 63.1 |  | | | 64.9 | | | *B* | | | 64.0 | | |  | | | 111.2 | | |  | | | 128.2 | | |  | | | 119.7 | | | |  |
|  | Average | **63.3** | **B^2^** | | | **65.2** | | | **A** | | | 64.3 | | |  | | | **112.8** | | | **B^2^** | | | **128.0** | | | **A** | | | 120.4 | | | |  |
| **CV** | | 2.43 | % | | | 0.64 | | | % | | | 1.70 | | | % | | | 7.70 | | | % | | | 1.50 | | | % | | | 5.23 | | | | % |

**: Differences between the averages followed by the same letter are not significant at P < 0.01 level. *: Differences between the averages followed by the same letter are not significant at P < 0.05 level. ^+,1,2^: Differences between the averages followed by the same letter are not significant at P < 0.01 level.
